# Communication Range Dynamics and Performance Analysis for a Self-Adaptive Transmission Power Controller †

**DOI:** 10.3390/s16050684

**Published:** 2016-05-12

**Authors:** Néstor Lucas Martínez, José-Fernán Martínez Ortega, Vicente Hernández Díaz, Raúl M. del Toro Matamoros

**Affiliations:** 1 Centro de Investigación en Tecnologías Software y Sistemas Multimedia para la Sostenibilidad (CITSEM), Universidad Politécnica de Madrid, Calle Alan Turing 3, 28031 Madrid, Spain; jf.martinez@upm.es (J.-F.M.O.); vicente.hernandez@upm.es (V.H.D.); 2 Centro de Automática y Robótica, Universidad Politécnica de Madrid, Carretera Campo Real Km. 0.2, 28500 Arganda del Rey, Spain; raul.deltoro@car.upm-csic.es

**Keywords:** self-adaptive systems, wireless sensor networks, transmission power control, connectivity, communication range

## Abstract

The deployment of the nodes in a Wireless Sensor and Actuator Network (WSAN) is typically restricted by the sensing and acting coverage. This implies that the locations of the nodes may be, and usually are, not optimal from the point of view of the radio communication. Additionally, when the transmission power is tuned for those locations, there are other unpredictable factors that can cause connectivity failures, like interferences, signal fading due to passing objects and, of course, radio irregularities. A control-based self-adaptive system is a typical solution to improve the energy consumption while keeping good connectivity. In this paper, we explore how the communication range for each node evolves along the iterations of an energy saving self-adaptive transmission power controller when using different parameter sets in an outdoor scenario, providing a WSAN that automatically adapts to surrounding changes keeping good connectivity. The results obtained in this paper show how the parameters with the best performance keep a k-connected network, where k is in the range of the desired node degree plus or minus a specified tolerance value.

## 1. Introduction

The Wireless Sensor and Actuator Networks (WSAN) are a well-known wireless communication technology with benefits that are becoming significantly important for solving upcoming societies challenges [1]. The WSAN reliability is strongly affected by unpredictable changes in the environment. A node transmitting always using its maximum power will show a reliability highly immunized to changes in the environment, but the node energy consumption will unnecessarily soar. Thus, a trade-off between energy consumption and communication reliability is required, as proposed in diverse strategies. For instance, in [2], Kotian *et al*. analyze four algorithms for a proactive transmission power control system, where the transmission power is tuned according to link quality predictions. Nodes must interchange information to measure link quality for making predictions. In [3], Mahmood *et al*. provide a survey on reliability protocol schemes based on retransmission and redundancy. For overcoming data loss, the use of data retransmission techniques and data redundancy is proposed, so that the destination can rebuild lost data from extra transmitted information. In [4], Djemili *et al*. propose two algorithms that adjust the node transmission power considering the distance to its two-hop neighbors and to the base station. In [5], Al-Bzoor *et al*. propose an adaptive power control routing protocol for underwater sensor networks. That protocol runs in two phases: first, the base station arranges the network so that nodes are clustered according to their distance to the base station, and second, nodes in a cluster must determine which one has the higher energy to become the cluster gateway to the base station. In [6], Ning *et al.* survey different routing protocols for underwater acoustic sensor networks, focusing on their energy consumption. Summarizing, routing protocols based on node location could reduce energy consumption, compared to those that do not locate neighbors, but increase node memory footprint and processor overhead. Conversely, location-free routing protocols provide worse network latency and increase overall network energy consumption, because of the cooperation among nodes to get network routes to the destination, but are gentle with node computational resources. In [7], Huang *et al*. discuss how to improve network resilience by modifying the network topology and/or the routing protocol. In [8], Kusy *et al*. show how radio diversity improves network reliability though there is a slight increase in the energy consumption. Nodes’ radio transceivers use dual widely-spaced radio frequencies through spatially-separated antennas. That approach only requires new hardware for the node (new radio transceivers and antennas) and does not imply any overload on the node processor or memory, as software or protocols already deployed are not changed. Nevertheless, there is an energy overload of 33%.

In a previous work [9], we propose a self-adaptive strategy, based on fuzzy control, which adapts each node transmission power to achieve an optimal number of neighbors (an optimal node degree). This optimal number of neighbors guarantees the node a high likelihood to reach any other node in the WSAN and only depends on the parameters of the specific WSAN: the deployment area and the number of nodes per m^2^. The node transmission power is dynamically adapted, and thus, the energy consumption is optimized.

**Figure 1:**
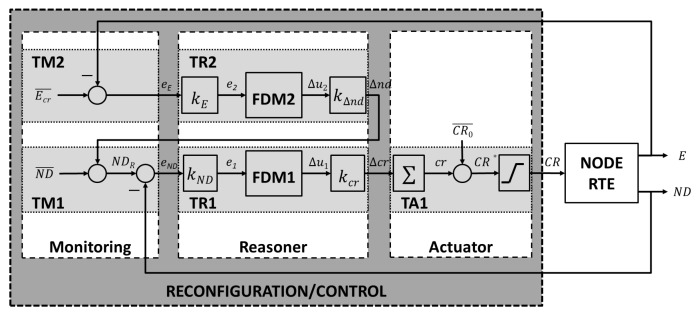
Control system design for self-adapting the Wireless Sensor and Actuator Networks’ (WSAN) nodes transmission power considering the number of neighbors and the battery level.

Such a self-adaptive strategy was carried out as described in another previous work [10], where the proposed fuzzy control system running in each node in a WSAN includes two feedback control loops as depicted in Figure 1. A primary feedback control manages the node transmission power considering both its real and targeted number of neighbors, as mentioned in the previous paragraph. A secondary feedback control loop was added to extend the node battery lifetime by managing the node targeted optimal number of neighbors considering the battery level. Whenever the node battery level drops below a critical value, the targeted number of neighbors is decreased, whereby the node transmission power is reduced and, thus, the node energy consumption.

In some of the above-mentioned related works, improvements in the WSAN energy consumption are carried out by means of new routing protocols, entailing an overload for the network. In others, the node must run algorithms for either making predictions or estimating node locations or distance to other nodes, which might not be accurate in real deployments and involve stressing node computational resources. However, the solution proposed in Figure 1, deeply described in the following sections, has no significant impact on the performance of existing WSANs, because it requires few extra computational resources in a node, as it implies running very simple algorithms, and does not require data interchange among nodes over the network, whereas the built-in routing protocol in the node provides accurate information about the number of neighbors, no matter the distance.

The rationale for the system in Figure 1 is available in [11,12,13]. Briefly, the probability of a WSAN being k-connected is the same as the probability of all WSAN nodes having a minimum number of neighbors equals to *k* and depends on the number of deployed nodes *n*, the node density *ρ*, defined as *n/A*, where *A* is the deployment area, and the nodes’ radio range *r*, as shown in Equation (1).

(1) $$P\left(k\right)={\left(1-\sum_{N=0}^{k-1}\frac{{\left(\rho \pi r^{2}\right)}^{N}}{N!}e^{-\rho \pi r^{2}}\right)}^{n}$$

Thus, for a concrete set of deployment parameters, an optimal number of neighbors for all network nodes can be estimated for guaranteeing that all of them have a good connectivity probability. At this juncture, the node target must be to manage its power transmission accordingly to keep the minimum number of neighbors estimated for its network always, as depicted in Figure 1, which also considers decreasing a node’s desired number of neighbors to extend its battery lifetime, though the probability of good network connectivity also decreases.

In addition, in [14], Huang *et al*. compare the fuzzy logic controller against other techniques for WSAN topology control, and the results from simulation highlight the fuzzy logic controller benefits. Additionally, in [15], Huang *et al*. compare fuzzy logic controllers obtained through a training dataset and fuzzy logic controllers based on heuristic if-then rules and membership functions. The former are preferred for WSAN deployments that can be accurately described by a mathematical model, and the latter is preferred when no mathematical model is available or accurate enough. For the approach analyzed in this paper, the latter kind of controller has been carried out.

This paper shows the results concerning the WSAN nodes’ connectivity and energy consumption from a real deployment, accomplishing the system in Figure 1. This paper’s novelty lies in the system simplicity. The approach is very easy to carry out and does not impose significant burdens to being deployed in existing WSANs. However, it includes some uncertainty, as a high probability of getting a k-connected network does not guarantee it is. Besides, the results in this paper come from a real deployment, not from simulations, like in most of the related previous works.

In the following sections, the implemented system’s inner details and its software design are discussed in Section 2 and Section 3, respectively. Section 4 describes the deployed experiments, and Section 5 analyzes and compares the results from those experiments. Finally, Section 6 presents the conclusions and depicts future works.

## 2. System Specification

This section provides a deeper view of the system in Figure 1. The contents of Subsection 2.1 have been published in [9,10], and they are summarized here for the sake of clarity and the reader’s convenience. Subsection 2.2 introduces and describes a set of rules that aim at avoiding the reasoner being triggered unnecessarily. That way, computational resources are saved, and the transmission power is not changed uselessly.

### 2.1. Overall System Description

In each feedback control loop in Figure 1, there is a decision-making function based on fuzzy logic (FDM1 and FDM2) that actually decides what to do at each moment.

Within the primary feedback control loop, the communication range (*CR*) (*i.e.*, transmission power) can be updated at a certain time instant *k* as follows:

(2) $$e_{ND}\left(k\right)=ND_{R}\left(k\right)-ND\left(k\right)$$

(3) $$e_{1}\left(k\right)=k_{ND}\cdot e_{ND}\left(k\right),\;\Delta cr\left(k\right)=k_{cr}\cdot \Delta u_{1}\left(k\right)$$

(4) $$\Delta u_{1}\left(k\right)=f_{DM1}\left(e_{1}\left(k\right)\right)$$

(5) $$\begin{array}{c} CR^{*}\left(k\right)=cr\left(k\right)+\bar{CR_{0}}\\ cr(k)=cr(k-1)+\Delta cr(k) \end{array}$$

(6) $$CR\left(k\right)=\left\{\begin{array}{cc} CR_{min}, & \;CR^{*}\left(k\right)<CR_{min}\\ CR^{*}\left(k\right), & \;CR_{min}\leq CR^{*}\left(k\right)\leq CR_{min}\\ CR_{max}, & \;CR^{*}\left(k\right)>CR_{min} \end{array}\right.$$

$\bar{CR_{0}}$ is the initial value of the communication range (*i.e.*, the initial transmission power) and $f_{DM1}$ represents the FDM1 function. The function input $e_{1}$ is the normalized node degree error, and the output $\Delta u_{1}$ is the normalized communication range variation factor. Furthermore, the value of CR is saturated between its minimum value $CR_{min}$ and maximum value $CR_{max}$. $k_{ND}$ and $k_{cr}$ are normalization or scale factors for the input and the output, respectively. Both factors can be calculated as:

(7) $$k_{ND}=1/\bar{ND},\;k_{cr}=\bar{\Delta cr}$$

where $\bar{ND}$ represents the nominal or desired value of the node degree when the battery has a critical energy level ($\bar{E_{cr}}$). Factor $\bar{\Delta cr}$ is the communication range variation rate.

The secondary feedback control loop tunes the desired value for the node degree according to the battery level and can be formalized as follows:

(8) $$e_{E}\left(k\right)=\bar{E_{cr}}-E\left(k\right)$$

(9) $$e_{2}\left(k\right)=k_{E}\cdot e_{E}\left(k\right),\;\Delta nd\left(k\right)=k_{\Delta nd}\cdot \Delta u_{2}\left(k\right)$$

(10) $$\Delta u_{2}\left(k\right)=f_{DM2}\left(e_{2}\left(k\right)\right)$$

(11) $$ND_{R}\left(k\right)=\bar{ND}+\Delta nd\left(k\right)$$

where $e_{E}$ is the difference between the battery critical level and the actual level and Δnd is the node degree variation factor. Besides, $f_{DM2}$ represents the FDM2 function where its input $e_{2}$ is the normalized or scaled value of $e_{E}$ and output $\Delta u_{2}$ is the scaled node degree variation factor. $k_{E}$ and $k_{\Delta nd}$ are scale factors for the input and the output, respectively. Both factors can be calculated as:

(12) $$k_{E}=1/\bar{E_{cr}},\;k_{\Delta nd}=\bar{\Delta nd}$$

where $\bar{\Delta nd}$ is the node degree variation rate.

In general terms, both loops can be seen as a function, which receives the actual values of ND and *E* as inputs, returning a new value of CR as output and requiring a set of parameters $\bar{P}$:

(13) $$\begin{array}{c} CR\left(k\right)=g\left(ND\left(k\right),E\left(k\right),\bar{P}\right)\\ \bar{P}=\left[\bar{CR_{0}},\bar{ND},\bar{E_{cr}},\bar{\Delta cr},\bar{\Delta nd},CR_{min},CR_{max}\right] \end{array}$$

where the complete parameters’ list is: the initial value of the communication range ($\bar{CR_{0}}$); the desired value of the node degree when the battery has a critical energy level ($\bar{ND}$); the critical energy level ($\bar{E_{cr}}$); the communication range variation rate ($\bar{\Delta cr}$); the node degree variation rate ($\bar{\Delta nd}$); the minimum and maximum value of the communication range ($CR_{min}$ and $CR_{max}$).

**Figure 2:**
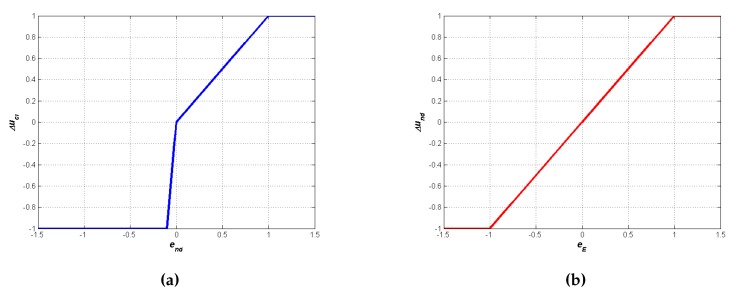
Fuzzy transfer functions. (**a**) FDM1; (**b**) FDM2.

The fuzzy transfer functions FDM1 and FDM2 are depicted in Figure 2a,b, respectively. Notice that FDM1 shows a larger slope for negative inputs than for positive values. When the FDM1 input is negative, the present number of neighbors is greater than the desired one $ND_{R}$, the node is wasting transmission power, because it does not need so many neighbors, and Figure 2a suggests to reduce the transmission power very quickly. Conversely, when the node needs more neighbors, the transmission power is increased slowly, approaching the accurate value for the transmission power little by little and avoiding energy wasting.

The system in Figure 1 complies with the MAPE-K [16] architecture, and three modules stand out:

- Monitoring: This receives as input the current state of the node and calculates the error variables.
- Reasoner: This executes the decision-making function.
- Actuator: This accomplishes the desired changes in the node.

Each of those modules are split into several tasks:

- *TM1*: This calculates the node targeted number of neighbors $ND_{R}$ by adding the WSAN optimal value $\bar{ND}$ and the change to be applied Δnd estimated by *TR2*, based on the battery level. Then, it samples the real number of neighbors ND using the node run-time environment, calculates the difference ($e_{ND}$) between the real and the targeted number of neighbors and decides if the *TR1* must be triggered.
- *TM2*: This samples the node’s real battery level *E* using the node run-time environment and computes the error $e_{E}$ as the difference between the critical battery level $\bar{E_{cr}}$ and the real battery level and then triggers *TR2* .
- *TR1*: This decides the change to be applied to the node communication range by means of its transmission power, so that the real and the targeted number of neighbors of the node become equal or quite similar. The *TR1* decision is driven by FDM1.
- *TR2*: This decides the change to be applied to the WSAN optimal number of neighbors $\bar{ND}$, thus influencing *TM1*. The decision-making process is driven by FDM2.
- *TA1*: This acts on the communication range by effectively establishing the node transmission power based on *TR1* output.

### 2.2. Rules

Rules presented and discussed in the following subsections have been defined to avoid unnecessary triggering of the reasoner.

#### 2.2.1. Activation Rule for *TR1*

The task *TR1* is triggered whenever necessary, and the rule that *TM1* considers for that is:

(14) $$\begin{array}{c} \mathrm{IF}\left(|e_{ND}\left(k\right)|-\xi_{ND}>0\right)\mathrm{OR}\left(ND\left(k\right)-ND_{min}<0\right)\mathrm{THEN}\\ \;\mathrm{EXECUTE}\;TR1\\ \;\mathrm{EXECUTE}\;TA1 \end{array}$$

This rule introduces $\xi_{ND}$ as a new parameter. The goal of this parameter is to define a tolerance or differential gap for the node degree error. This gap is necessary in order to reduce the oscillations due to continuous changes in the error sign. The oscillations can also be originated because the node degree reference value cannot be achieved due to the sensitivity of the node degree with respect to the variation in the communication range. The value of $\xi_{ND}$ is provided by:

(15) $$\xi_{ND}=\alpha_{\xi_{ND}}\cdot \Delta CR_{min}$$

where $\alpha_{\xi_{ND}}$ is a positive non-zero arbitrary value, and $\Delta CR_{min}$ is the minimum possible change in the communication range (*i.e.*, the transmission power).

This tolerance value provides a margin when evaluating the node degree error and is fixed for the controller lifetime. Therefore, it cannot prevent oscillations caused by unexpected changes in the network.

#### 2.2.2. Oscillations Rule for *TR1*

The tolerance factor $\xi_{ND}$ provides a good static approach to avoid oscillations. For dynamic WSANs, where the number of available neighbors can change at any moment and for whatever reason, other approaches should be taken into consideration. In this case, we have defined an oscillation rule that avoids the triggering of task *TR1* in certain conditions that can cause an oscillation. Table 1 provides a summary of the analysis performed in the definition of this rule.

On the left of Table 1 we can see the value of the previous iteration for Δcr. In a linguistic way the possibilities for the change represented by Δcr are negative big (NB); negative small (NS); no change (ZV); positive small (PS); and positive big (PB). These five possibilities are then checked with the previous and actual values of $e_{ND}$.

Then, the table can be read as follows: *If the actual error on the node degree $\left(e_{ND}\left(k\right)\right)$ is negative and the actual node degree (ND(k)) is greater than the adjusted desired node degree, then if there has been a previous small positive change on the communication range (Δcr(k-1)) and the previous error on the node degree $\left(e_{ND}\left(k-1\right)\right)$ was positive, then no change must be done in the communication range*.

As previously described, a negative error on the node degree should cause a negative change on the communication range; a positive error should cause a positive change; and no error should not cause any change at all. Table 1 introduces a couple of complementary situations that can cause an oscillation and that were not expected by the system. These two complementary situations are:

- The node has now a negative error for the node degree, meaning that it has more neighbors than desired. In the previous iteration, the error was positive, meaning that it had fewer neighbors than desired. Furthermore, the change in the communication range was the minimal. Thus, the reasoner will decide to change the communication range again, as the current error is beyond expected limits. However, we can foresee that even the minimal change will put the node into the previous situation, when it had fewer neighbors than desired. Thus, there is a high chance that if the change is made, in the next iteration, the error on the node degree will be positive, causing an oscillation. Therefore, in this case, no change should be made. The complementary is also valid.
- The node has now a negative error for the node degree, meaning that it has more neighbors than desired. In the previous iteration, the error was also negative, and no change was applied to the communication range. This means that in the previous iteration, the system decided not to update the communication range to prevent an oscillation (this is the only way to reach this situation). In this case, the reasoner will decide to change the communication range, as the current error is beyond the limits. However, if the previous iteration tried to prevent an oscillation and nothing has changed from it, then there is still no need to change the communication range. Additionally, again the complementary is also valid.

The following rule has been defined as a result of the analysis of Table 1:

(16) $$\begin{array}{c} \mathrm{IF}\\ \;\{sign\left(e_{ND}\left(k\right)\right)=sign\left(e_{ND}\left(k-1\right)\right)\mathrm{AND}\Delta cr\left(k-1\right)=0\}\\ \;\mathrm{OR}\\ \;\{e_{ND}\left(k\right)<-\xi_{ND}\mathrm{AND}e_{ND}\left(k-1\right)>\xi_{ND}\mathrm{AND}|\Delta cr\left(k-1\right)|=\Delta cr_{min}\}\\ \;\mathrm{OR}\\ \;\{e_{ND}\left(k\right)>\xi_{ND}\mathrm{AND}e_{ND}\left(k-1\right)<-\xi_{ND}\mathrm{AND}|\Delta cr\left(k-1\right)|=\Delta cr_{min}\}\\ \mathrm{THEN}\\ \;\mathrm{DO}\mathrm{NOTHING}\\ \mathrm{ELSE}\\ \;\mathrm{EVALUATE}\;ACTIVATION\;RULE\;FOR\;TR1 \end{array}$$

The rule in Equation (16) must precede the rule in Equation (14).

#### 2.2.3. Activation Rule for *TR2*

Task *TM2* calculates the difference $e_{E}$ between the battery critical level and its actual level. It also can control the execution of task *TR2*. The goal is to avoid the execution of this reasoning function when its output will be saturated and, therefore, does not perform any unnecessary computation of this function to obtain a new output.

According to the definition of the decision-making function, the activation rule for *TR2* can be defined as:

(17) $$\begin{array}{c} \mathrm{IF}\;\left(e_{E}\left(k\right)-\frac{1}{k_{e}}\right)\leq 0\;\mathrm{THEN}\\ \;\mathrm{EXECUTE}\;TR2\\ \;\mathrm{EXECUTE}\;TR1\\ \;\mathrm{EXECUTE}\;TA1 \end{array}$$

#### 2.2.4. Saturation Rule

If $ND_{R}$ has been updated or the primary controller is not activated because the node degree error $e_{ND}$ is within the acceptance region and also the controller output is saturated, then the output of the controller must be driven out of the saturation value. The appropriate new value for the controller output is unknown because the controller does not contain a model that relates its output with the node degree error, and also, this relation is non-linear and non-injective. The rule to achieve this is as follows:

(18) $$\begin{array}{c} \mathrm{IF}\\ \;\{|e_{ND}\left(k\right)|-\xi_{ND}\leq 0\}\\ \;\mathrm{AND}\\ \;\{CR_{f}\left(k\right)-CR_{max}\geq 0\}\\ \;\mathrm{AND}\\ \;\{\left[ND_{R}\left(k\right)\neq ND_{R}\left(k-1\right)\right]\mathrm{OR}\left[e_{ND}\left(k\right)<e_{ND}\left(k-1\right)\right]\}\\ \mathrm{THEN}\\ \;e_{ND}\left(k\right)=-\left(\xi_{ND}+\Delta CR_{min}\right) \end{array}$$

The introduction of a small disturbance to the node degree error forces the activation of the primary control function. This will force the controller to decrease the output trying to correct the error. This rule introduces the new parameter $CR_{f}$, which is the value of CR being filtered to avoid punctual values of CR saturated, according to Equation (19).

(19) $$CR_{f}\left(k\right)=\frac{1}{2}\left[CR\left(k\right)+CR\left(k-1\right)\right]$$

## 3. System Design

**Figure 3:**
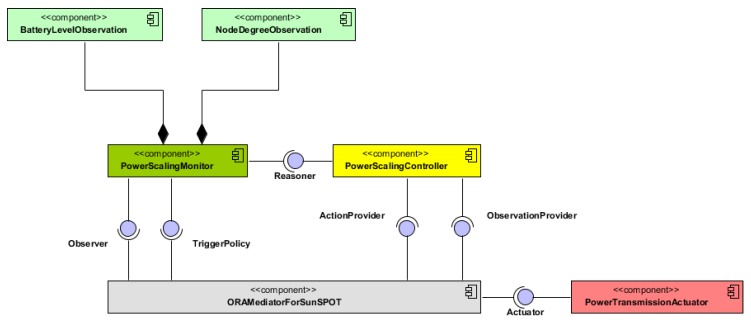
Components for the controller following a MAPE-K approach.

The Figure 3 depicts the UML (Unified Modeling Language) component diagram for the system in Figure 1. This figure proposes a general software design for accomplishing self-adaptive systems based on MAPE-K [16]. It is also based on the one fully described in [17] customized for the SunSPOT platform, the deployment platform.

The component ***ORAMediatorForSunSPOT*** enables the interaction among the rest of the components. It behaves as a broker decoupling observers, triggers, reasoners and actuators. Therefore, all of the components required for the reasoning engine must be previously registered on it. It also provides the observations and actions to either the reasoner or the monitor whenever they are required.

**Figure 4:**
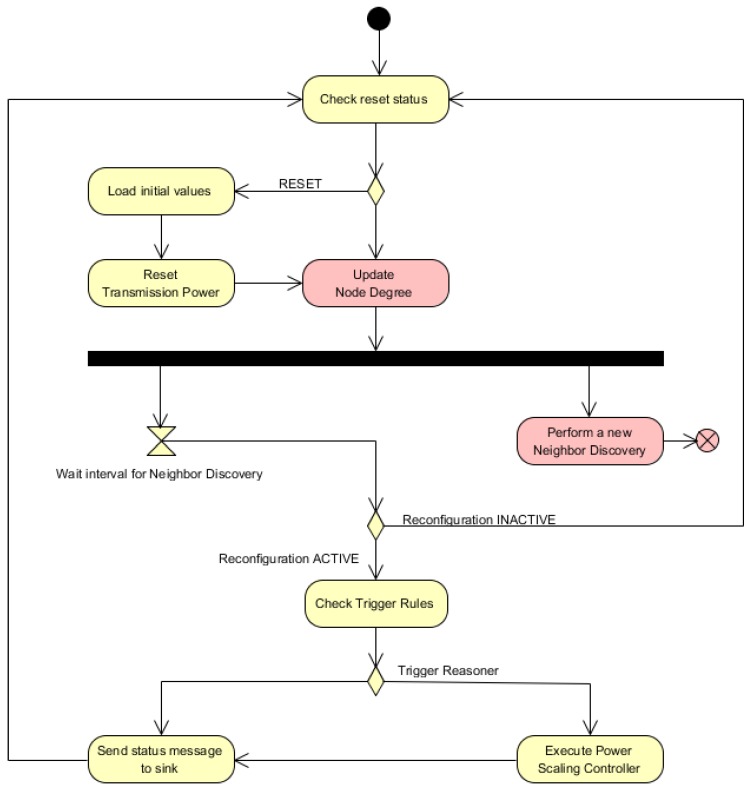
Activity diagram for the component ***PowerScalingMonitor***.

The components ***PowerScalingMonitor***, ***BatteryLevelObservation*** and ***NodeDegreeObservation*** in Figure 3 shape the monitoring module in Figure 1. The components ***BatteryLevelObservation*** and ***NodeDegreeObservation*** measure respectively the battery level and the number of neighbors of a node. The component ***PowerScalingMonitor*** monitors at a specific rate the number of neighbors and the battery level of a node. With the updated values of the sensed parameters, this component then evaluates the rules discussed previously and, if required, triggers the reasoner represented by the ***PowerScalingController*** component. The activity diagram for this component is illustrated in Figure 4.

**Figure 5:**
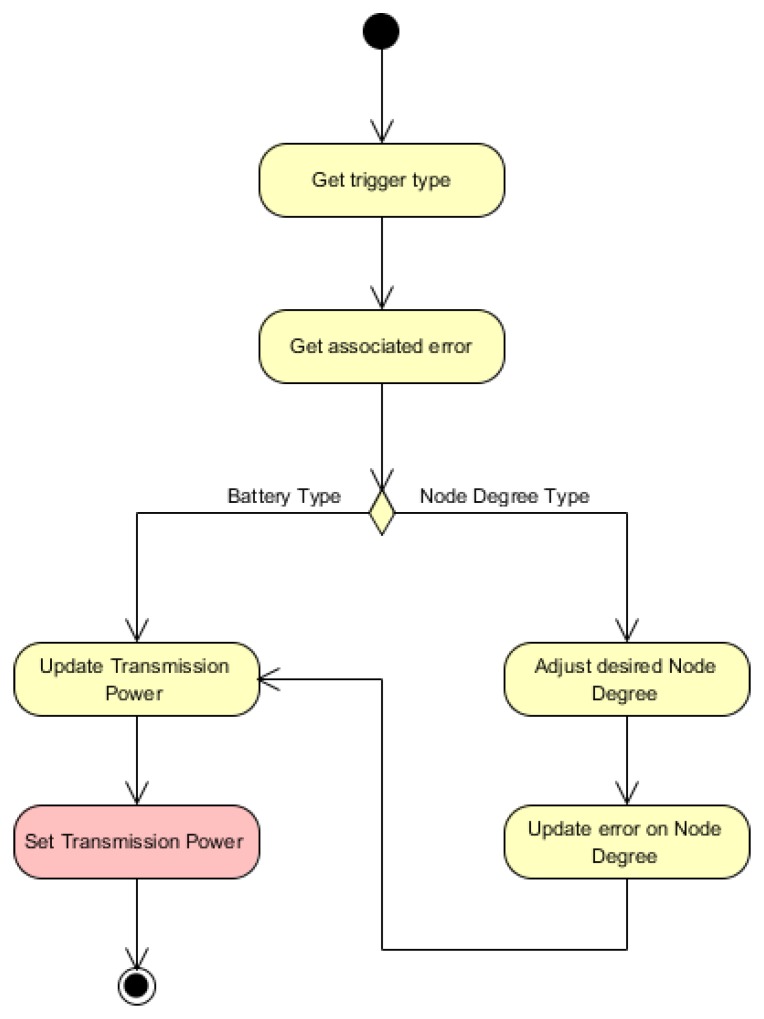
Activity diagram for the component ***PowerScalingController***.

The ***PowerScalingController*** component matches the reasoner module from Figure 1, including both task *TR1* and *TR2*. Therefore, it implements the execution of the fuzzy decision-making functions to evaluate the error, either the node degree error $e_{ND}$ or the battery level error $e_{E}$. The activity diagram for this component is exposed in Figure 5.

### 3.1. Neighbor Discovery Protocol

As mentioned in the Introduction section, the node number of neighbors could be usually obtained from the built-in routing protocol. Thus, no extra components are needed for discovering the node neighbors, encouraging this approach to be readily embedded in existing WSAN solutions.

However, that is not the case for the deployment platform that is to be analyzed in this paper. The SunSPOT library provides two routing protocols: *Ad hoc* On-demand Distance Vector (AODV) [18] and Link Quality Routing Protocol (LQRP) [19]. LQRP is indeed a modified version of AODV, where the next hop in a route is selected by the link quality indicator (LQI) instead of the number of hops. Both of these protocols keep a neighbor list. However, its information is only updated on two occasions:

- Whenever the validity of an entry expires.
- Whenever a new route request is performed.

This is not enough for the purpose of the self-adaptive controller described so far, as the information will not be updated unless a new route discovery is requested.

Therefore, a neighbor discovery protocol has been carried out. This does not replace the built-in routing protocol in the SunSPOT platform. Indeed, both protocols run in parallel.

The neighbor discovery protocol in question is quite simple, conceived just for testing the adaptability of the controller. In any case, there is a whole area of study focused on the exploration of neighbor discovery protocols for wireless sensor networks, either passive, active or both [20,21,22,23].

**Figure 6:**
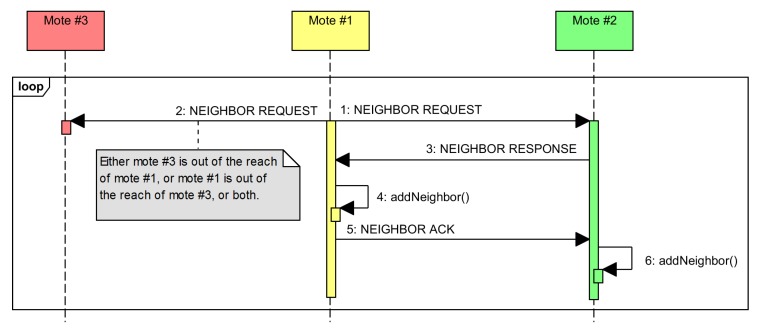
Sequence diagram for the neighbor discovery protocol.

The accomplished neighbor discovery protocol is an active three-way one, as shown in Figure 6. The node that wants to update its neighbor list starts the handshake by broadcasting a NEIGHBOR REQUEST message. The nodes that receive the message issue a unicast NEIGHBOR RESPONSE message. Upon receiving this message, the originating node knows that it is able to reach the responding one and receive the messages, so it can be considered as a neighbor and, thus, added to the list. It then also issues a unicast NEIGHBOR ACK message to acknowledge the other node as a neighbor. At this point, the second node knows that it has been able to reach the originating one, as well as getting its messages. Therefore, it can also be added to its own neighbor list.

**Figure 7:**
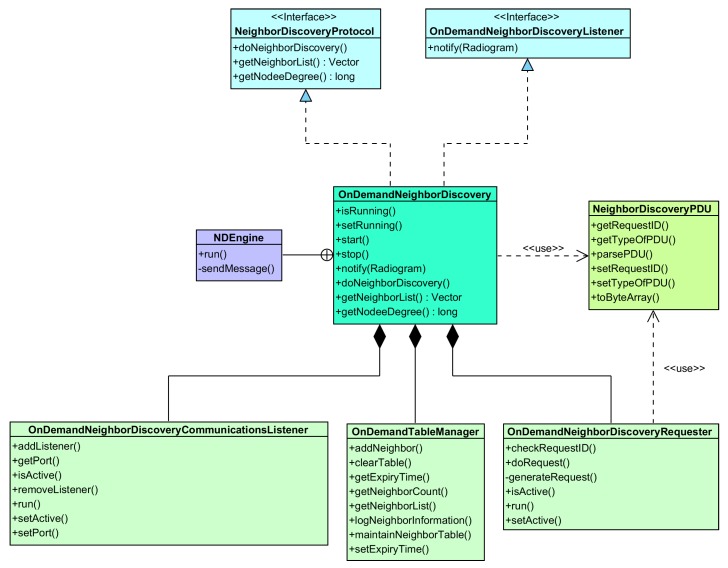
Class diagram for the neighbor discovery protocol.

The class diagram for the neighbor discovery protocol is illustrated in Figure 7.

## 4. Materials and Methods

### 4.1. Equipment Used

**Figure 8:**
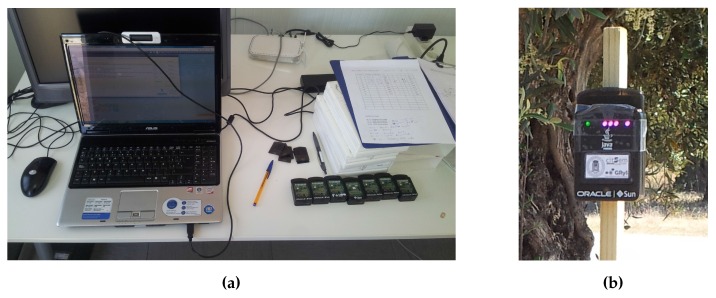
Equipment used in the experiments. (**a**) Computer and standalone sensor nodes; (**b**) deployed sensor node.

A typical WSAN was deployed for the performance tests, consisting of eight standalone SunSPOT sensor nodes [24] and one SunSPOT base station connected to the computer and acting as a sink for collecting the results from the experiments. The equipment used is shown in Figure 8, and the source code for the developed controller has been released as open source and is available at GitHub [25].

#### 4.1.1. Sensor Nodes

The sensors used for the experiments were Oracle Sun Small Programmable Object Technology (SunSPOT) devices. The SunSPOT platform provides a framework for rapid prototyping of WSAN applications using J2ME CLDC API v1.1.

SunSPOT devices are modular, so their hardware capabilities can be extended by the addition of extra boards. The typical SunSPOT sensor configuration is composed of two boards: the main board and the sensor board. To operate as a standalone, all SunSPOT sensors are equipped with a 3.7-V battery with a nominal capacity of 770 mAh. The main board also provides wireless communication capabilities through a CC2420 module using an inverted-F antenna. This enables the SunSPOT devices to communicate with other 802.15.4-capable devices that use the 2.4-GHz band.

These sensor devices can be programmed using a restricted Java subset for a specific Java Virtual Machine, known as Squawk [19]. This micro edition virtual machine for embedded systems and small devices was designed to have a small footprint with minimal external dependences [26,27].

To ease the programming of applications for the Internet of Things, the SunSPOT platform also includes its own set of extra libraries to be run on top of Squawk. The SunSPOT software development kit (SDK) gives access to the communications and to the resources on the sensor board. The last stable version of the SunSPOT SDK was released in November 2010 as Version 6.0 and codenamed “Yellow”. This version has some issues when trying to check the available capacity of the battery, returning random values. This issue was solved in the last developers version of the SDK, released in May 2011 as Version 7.0 and codenamed “Teal”. We have updated the sensor nodes used in the experiments to this last version so we can make use of the correct information about the available battery capacity.

The sink node is just like any other SunSPOT device without the sensor board and the battery. It allows a computer to interact with the sensors. We have developed a remote control application that allows us to control the execution of the experiments and to collect the received information from the deployed sensors. The sensors themselves also store information related to the operation of the self-adaptive system, the battery status and their node degree in order to compare the results from the experiments.

### 4.2. Experimental Setup

The experiments were performed in an open area at the facilities of the Centro de Automática y Robótica of the Consejo Superior de Investigaciones Científicas in Arganda del Rey (Spain). The base station was fixed to the outside of a stall at an approximate height of 1.5 m. The deployment of the nodes can be decided either by deterministic or random mechanisms [28,29]. The connectivity is related to the sensing coverage [30], and in a deterministic deployment, the transmission power for each node can be estimated beforehand to get a k-connected network. Therefore, we have chosen a random deployment where our proposed self-adaptive system can be used to dynamically adjust the transmission power. This adjustment is used to reach a steady state where the connectivity can guarantee a good packet delivery rate while keeping the energy consumption as low as possible. The only restriction we have considered for the locations is that the farthest node from the base station should be at a distance that maximizes the chances of using the multi-hop capabilities of the underlying routing protocol.

**Figure 9:**
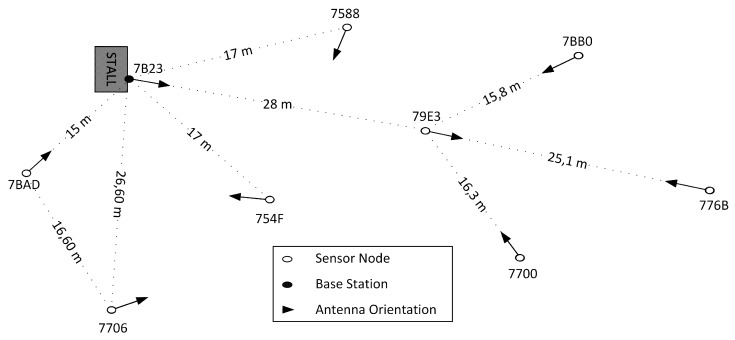
Deployment, distances and nodes orientation.

A scheme of the deployment is shown in Figure 9. The schema includes information about the identities of each node and the distances between them. The heights for the sensor nodes were above one meter in all cases, but one, which was closer to the ground at a height of about 40 cm.

Figure 9 also shows the orientation of the antennas, as this factor along with the irregularity in their radiation patterns introduces a non-negligible phenomenon that has an impact in the strength of the received signal (RSSI) [31]. The orientations were also random.

The scenario was in an isolated area, so no radio interferences were expected. The radio channel used was the default Channel 26. The transmission power for this channel is restricted to the range from −3 dBM to −32 dBM [32]. All of the sensor nodes start each experiment at the lowest available transmission power. At each iteration of the control loop, the self-adaptive system readjusts the transmission power according to the number of neighbors. The interval of the monitoring loop was set to 20 s.

The parameters being evaluated were:

- $ND_{R}$ with the possible values of two and three.
- $\xi_{ND}$ with the possible values of zero and one.
- $k_{CR}$ with the possible values of one and three.

The $E_{CR}$ parameter was fixed to a value of 150 mAh.

As this was a reduced set of values, it was considered worthy to perform a factorial design of the experiments. Additionally, two control experiments were included using a fixed transmission power with no self-adaptive system running, but in equivalent conditions. That is, the rest of the activities performed by the nodes, like the discovery of their neighbors and the collection and report of sensed data, remained the same. The two control experiments provided a set of reference values to compare with those obtained from the use of the self-adaptive system. Table 2 summarizes the values used in each experiment, where e01 and e02 refer to the two control experiments already mentioned. It also collects the results obtained in [10] related to the energy consumption and the connectivity.

## 5. Results

### 5.1. Communication Range Dynamics

A self-adaptive system will try to keep itself in a steady state by executing the necessary actions to achieve its predefined functionality regardless of external perturbations [33]. The goal of the self-adaptive system is therefore to reduce the impact of the external perturbations, or stimuli, in the normal operation of the system. In our case, the predefined functionality of the proposed fuzzy control-based self-adaptive system is to keep the network connectivity of a wireless sensor network using a probabilistic approach based on the number of neighbors, or node degree, of each node. The only action that a node can do at any iteration of the control loop is to increase or decrease its communication range by means of acting on its own transmission power. We can say then that the system has reached a steady state when there is no change in the transmission power of the node or even when the change is so small and smooth that it keeps the network connectivity over a minimum performance limit.

**Figure 10:**
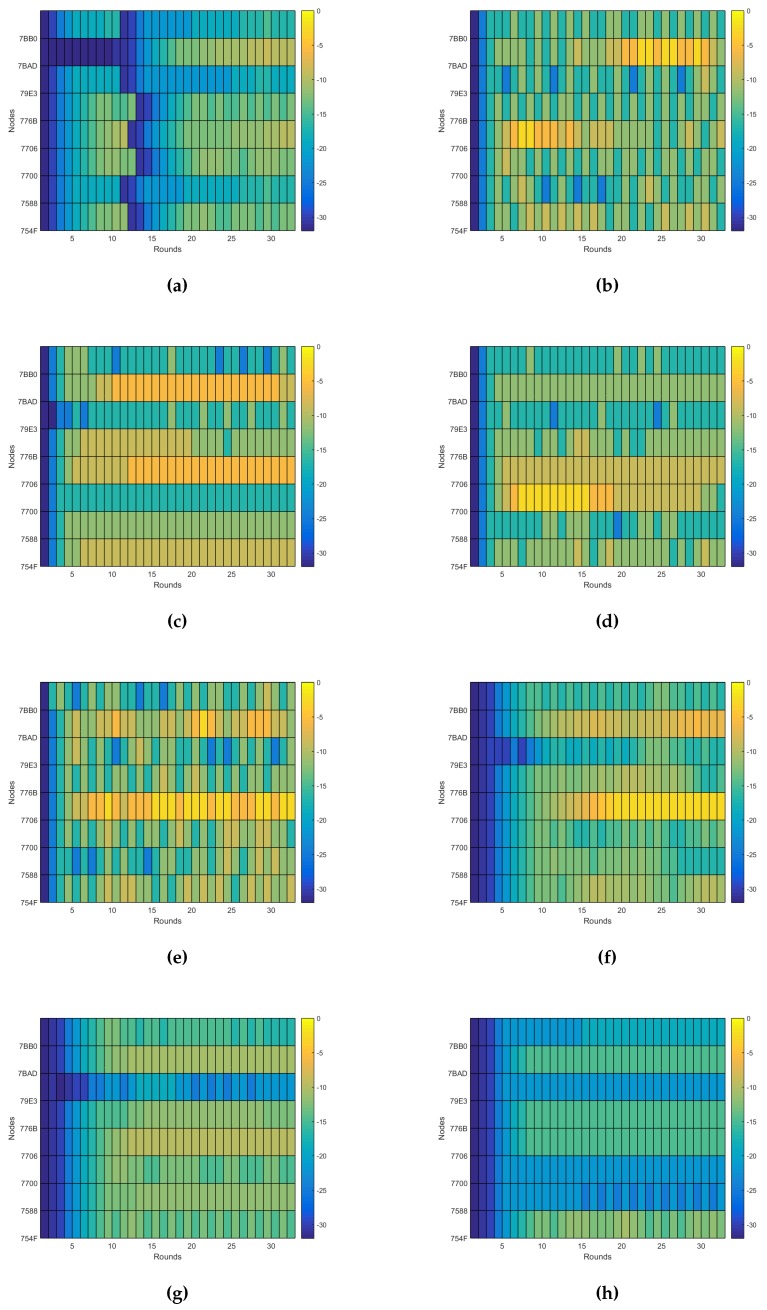
Transmission power dynamics. (**a**) Experiment e03; (**b**) Experiment e04; (**c**) Experiment e05; (**d**) Experiment e06; (**e**) Experiment e07; (**f**) Experiment e08; (**g**) Experiment e09; (**h**) Experiment e10.

**Figure 11:**
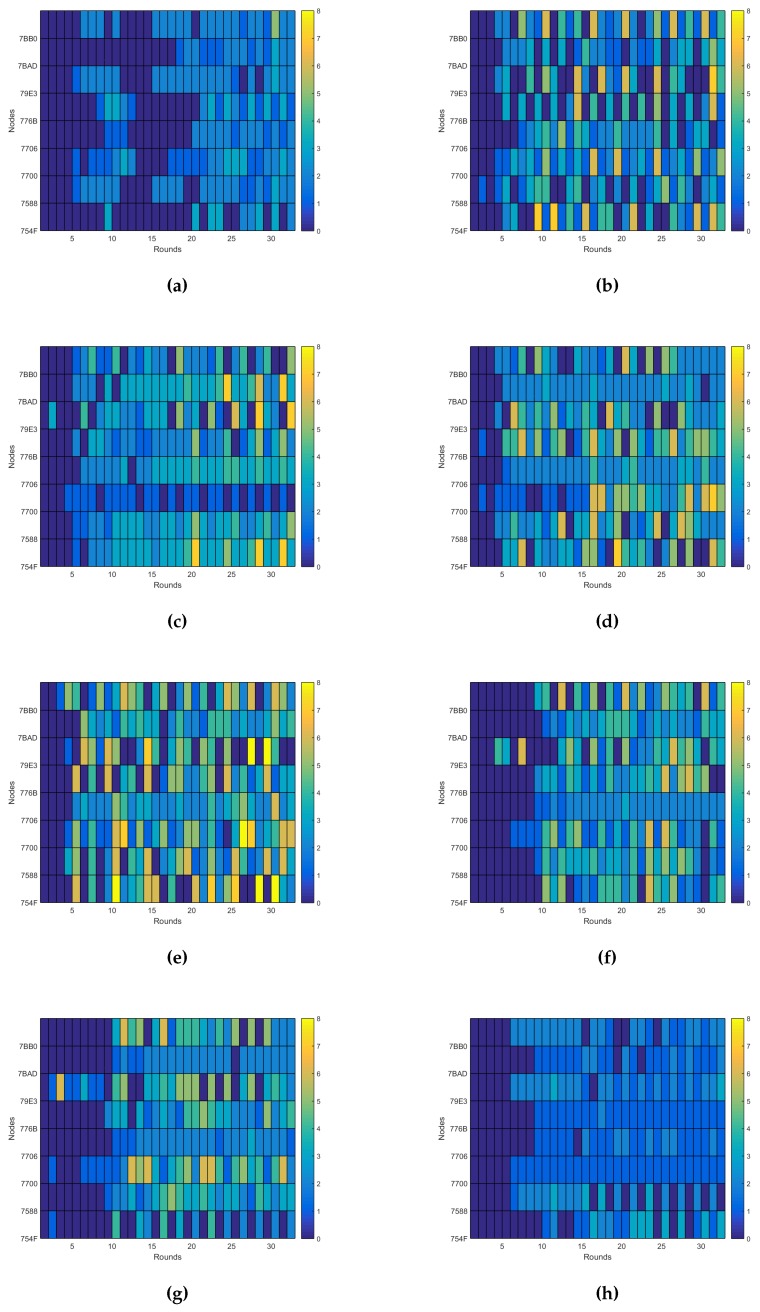
Node degree dynamics. (**a**) Experiment e03; (**b**) Experiment e04; (**c**) Experiment e05; (**d**) Experiment e06; (**e**) Experiment e07; (**f**) Experiment e08; (**g**) Experiment e09; (**h**) Experiment e10.

**Figure 12:**
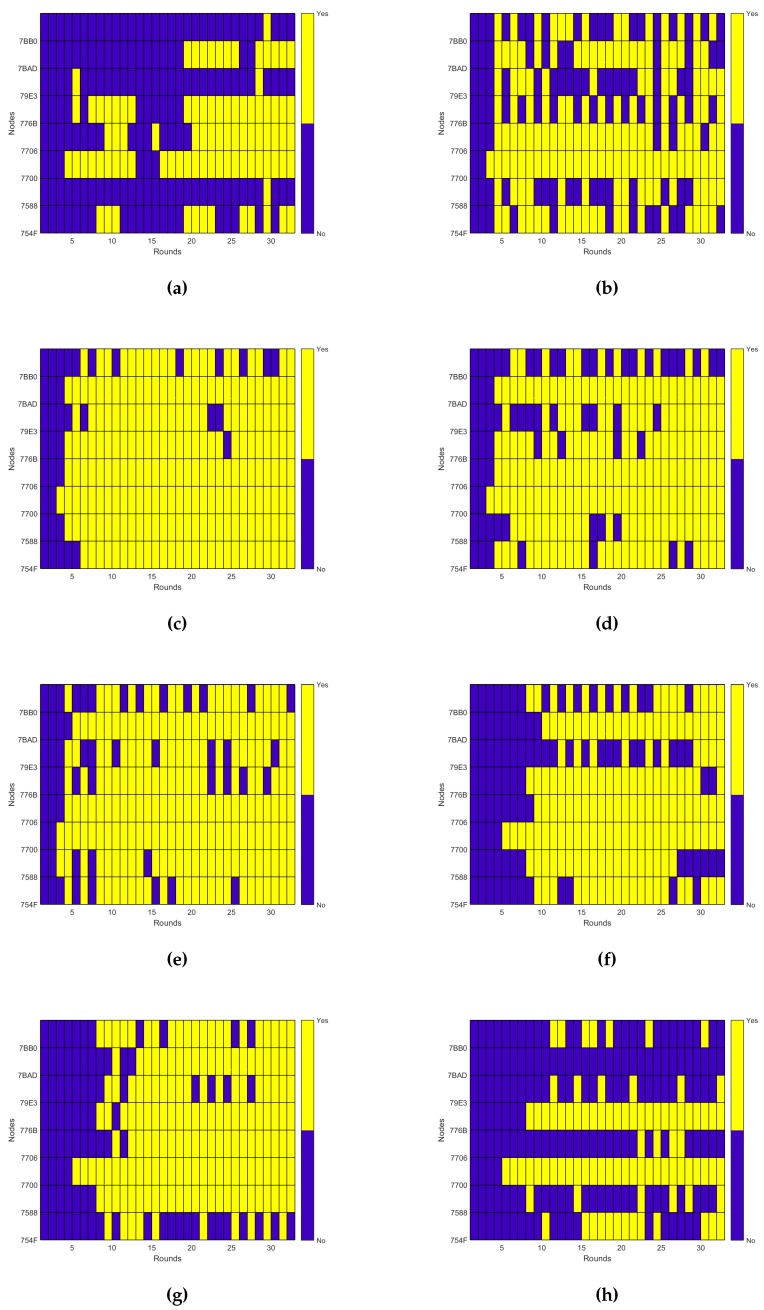
Packet delivery dynamics. (**a**) Experiment e03; (**b**) Experiment e04; (**c**) Experiment e05; (**d**) Experiment e06; (**e**) Experiment e07; (**f**) Experiment e08; (**g**) Experiment e09; (**h**) Experiment e10.

Thus, to evaluate the performance of the system beyond the energy consumption and the connectivity, as analyzed in [10], here, we are going to evaluate its dynamical behavior. The dynamical behavior allows us to know how much effort is required for a self-adaptive system to reach its steady state. We can talk then about two main measures: the total number of actions performed by the self-adaptive system during the experiments and how fast it reaches a steady state, expressed as the number of iterations of the control loop required. As described previously, the only action the system does is over the transmission power, so these measurements are calculated just taking into account the changes in the transmission power, as shown in Figure 10. In any case the dynamics relative to the node degree and the connectivity are also shown in Figure 11 and Figure 12, respectively, as they provide additional information of interest.

The figure of merit calculated for the transmission power dynamics is obtained using Equation (20), where *i* represents the node and *k* the iteration. Therefore, this equation provides us with the total number of actions done on the transmission power for all of the nodes during the running of the experiment.

(20) $$J_{d}=\sum_{i=0}^{N}\sum_{k=0}^{M}\chi_{i}\left(k\right)$$

The results of applying Equation (20) to the obtained data from the experiments is shown in Table 3.

There are several conclusions we can derive from both the figures and the values listed in Table 3. The first and easiest thing we can conclude is that Experiment e05 has the best performance regarding the number of actions on the transmission power, as it uses just only 58 changes during the experiment and reaches a good enough connectivity after just six iterations of the control loop, achieving a 92.48% PDR in the seventh iteration. Indeed, as we will see later, the expected delivery rate of this experiment reaches a 99% after the 10th iteration.

Another thing we can observe is that although the dynamics estimation provides interesting information, it has to be considered along with other performance measurements. For instance, Experiment e07 has one of the worst dynamics figures, with 205 changes of the transmission power during the experiment, but it has valid connectivity results, reaching an expected delivery rate of 90% in its 16th iteration, with connectivity values over the 80% PDR from the 10th iteration, as seen in Figure 12e. The same happens the other way. Experiment e10 has the third best figure for the transmission power dynamics, but due to a bad choice of the configuration parameters, it provides a really bad connectivity, never reaching even an expected delivery of 50% of the messages transmitted, as seen in Figure 12h.

Thus, we cannot use just the dynamics estimation to evaluate the performance of the system. However, it still provides good complementary information to other performance measurements, like the energy consumption and the connectivity performance explored in [10]. It also produces other valuable information, like the evidence that the node degree is different for each node even when the network has reached a steady state. This can be observed when comparing Figure 10c and Figure 11c. This allows us to reformulate the question proposed in [34] about the adjustment of the desired node degree for each node according to its location in the network. Moreover, the node degree also changes when there is no change in the transmission power. For instance, in the aforementioned figures, we can observe that there is no change in the transmission power from Round 11 to Round 16 for Experiment e05. However, when observing the same interval for the node degree dynamics, we can see that the node degree changes among rounds. Additionally, this occurs without losing connectivity, as shown in Figure 12c. Of course, this resilience to the changes in the node degree is due to the use of the tolerance parameter $\xi_{ND}$, but it also illustrates that events external to the nodes affect the node degree. Therefore, the question that remains is if there is another way to solve the unexpected changes in the node degree beyond the use of a tolerance factor.

### 5.2. Analysis of the Effect of the Parameters on the System’s Performance

Once all of the figures of merit have been obtained, we can analyze the effect of each parameter in the outcome of the fuzzy control-based self-adaptive system. This final analysis is performed applying Equations (21) and (22) to the values obtained in the experiments in correlation to the grouping of the parameters’ set.

(21) $$SN_{p,v}=\frac{\sum_{n=0}^{N_{v}}J_{p,v}}{N_{v}}$$

(22) $$\Delta J_{m}=max\left(SN_{p,v}\right)-min\left(SN_{p,v}\right)$$

For instance, the estimation of the effect of each configuration parameter on the energy consumption, using the figures previously published in [10], is shown in Table 4. In our case, each configuration parameter can have two potential values. The $ND_{R}$ can be either two or three; the $\xi_{ND}$ can be zero or one; and the $k_{CR}$ can be one or three. Therefore, in this case, the first row, corresponding to the parameter level $SN_{p1}$, shows the sum of the figure $J_{e}$ values when the first value of the configuration parameters $ND_{R}$, $\xi_{ND}$ and $k_{CR}$ is used, divided by the number of times it is used (in this case, four). The second row shows the same for the second possible value for each configuration parameter. The final row shows the result of applying Equation (22) to the previous rows. The greatest value in this row identifies which parameter has the greatest effect on this figure of merit. In this case, the tolerance represented by $\xi_{ND}$ is the parameter that, according to this estimation, has the greatest effect on the energy consumption for the whole network, representing 47.02% of the total estimation of the effect. The $k_{CR}$ factor represents 36.08%, and the lowest impact on the energy consumption is represented by the $ND_{R}$, with 16.90% of the total estimation.

The same procedure is used to estimate the effect of each parameter on the connectivity, using again the figures from [10], as shown in Table 5. This time, the configuration parameter more relevant to the connectivity is the scaling factor $k_{CR}$, with 61.06% of the total estimation. The next parameter in relevance is the $ND_{R}$, with a 37.69% representation. Additionally, the smallest impact on the final result is due to the tolerance parameter $\xi_{ND}$, with 1.24%.

Finally, we make the proper estimation for the dynamics presented in this paper, focusing just on the transmission power dynamics, as that is the only dynamic on which the self-adaptive system acts. The results are shown in Table 6, and the tolerance represented by $\xi_{ND}$ is the parameter that has the greatest impact on the transmission power dynamics, representing 78.57% of the total estimation. Next follows the $ND_{R}$ parameter with 18.65%, and the smallest impact is caused by the $k_{CR}$ factor, with 2.78% representation.

As a summary, we can conclude that the parameter $\xi_{ND}$ is the most relevant for the proposed fuzzy control-based self-adaptive system, being the one with higher impact on the energy consumption and the communication range dynamics, although it has the lowest effect on the connectivity. Indeed, its overall contribution to the system’s performance represents 42.28%, while the $k_{CR}$ parameter contributes with 33.31%, and finally, the $ND_{R}$ parameter contributes with 24.41%.

### 5.3. Cost Estimation

The cost estimation allows us to evaluate which configuration of the system is better for a given necessity in the explored scenario. Equation (23) represents the cost function used in this analysis, where $w_{e}$ and $w_{c}$ are the weights assigned to the energy consumption and the connectivity, respectively, and $\bar{J_{e}}$ and $\bar{J_{c}}$ are the normalized values for the figures of merit for the energy consumption and the connectivity obtained in [10]. This cost estimation does not include the communication range dynamics, as its figures do not show a correlation to the system’s performance, as we have discussed in the previous section.

(23) $$J=w_{e}\bar{J_{e}}+w_{c}\bar{J_{c}}$$

The normalized values of $\bar{J_{e}}$ and $\bar{J_{c}}$ are obtained using Equation (24), where *x* refers to the specific figure, either *e* for energy or *c* for connectivity, and *y* is the experiment for which the estimation is being calculated, while $max(J_{x})$ is the maximum value for the figure of merit for all of the experiments.

(24) $$\bar{J_{x}}=\frac{J_{x,y}}{max(J_{x})}$$

Once we have the values for $\bar{J_{e}}$ and $\bar{J_{c}}$ for each experiment, we can just assume that the relation between the weights for the connectivity and the energy consumption follows Equation (25).

(25) $$w_{c}=1-w_{e}$$

**Figure 13:**
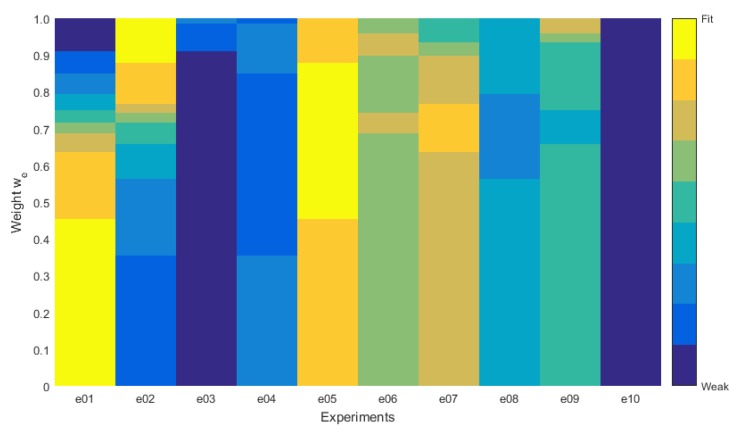
Map of minimums for cost estimation.

Then, we can assign a value from zero to one to the weight for the energy consumption to see how each experiment has behaved. The lowest value of the cost estimation indicates the best performance for the associated weights. Additionally, if we map all of the obtained results for the range assigned to both weights, we will obtain the result shown in Figure 13. This map represents how well a parameter set fits according to the assigned weights.

The conclusion to the cost estimation is that when the energy consumption is the least relevant condition, up to 45.5% (or the connectivity is the most relevant condition from 54.5%), we should use a fixed transmission power set to the maximum. Additionally, when the energy is the most relevant condition, from 87.8% (or the connectivity is the least relevant condition up to 12.2%), we should still be using a fixed transmission power, but in this case set to a medium value. Additionally, the most important thing, when the energy consumption has a relevance between 45.5% and 87.8%, we should use the fuzzy control-based self-adaptive system with the configuration from Experiment e05. Of course, these results are valid for the experiments we have made. Additionally, they illustrate that there is a range of relevance for either the energy consumption and the connectivity where the use of a self-adaptive system provides better results than using fixed transmission power.

### 5.4. Additional Conclusions

**Figure 14:**
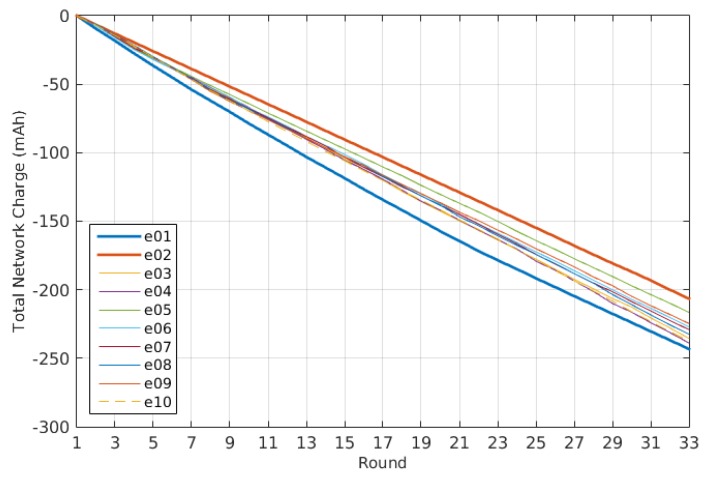
Total network discharge rate comparison.

**Figure 15:**
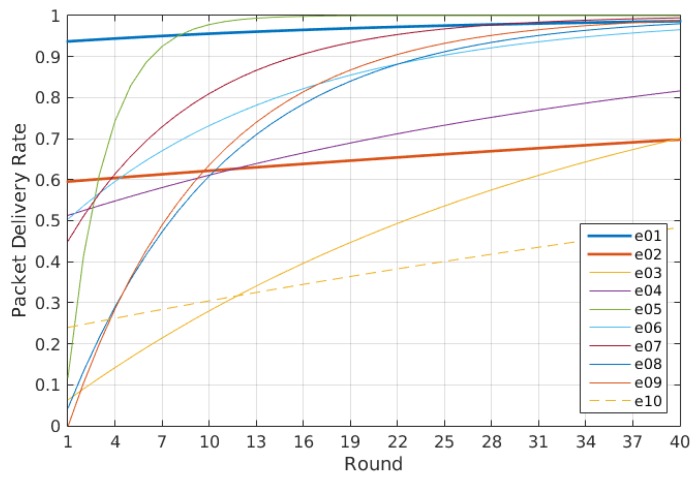
Network packet delivery ratio.

As Figure 14 shows, the minimum network energy consumption corresponds to Experiment e01, while the maximum corresponds to Experiment e02. In both cases, the transmission power was set to a fixed value. The first was set to a medium one, and the second to the maximum available one. In Figure 15, we can compare also the results for the connectivity as an expression of the tendency curves for the packet delivery ratio (PDR) for the whole network as presented in [10]. In this case, the situation is reversed: the maximum transmission power, as expected, provides a 99% PDR, while the medium transmission power merely reaches 70% PDR.

Therefore, the use of the self-adaptive transmission power depends on the importance assigned to the energy consumption and the connectivity, as discussed previously regarding the cost estimation. From the experiments performed to evaluate the performance of the proposed self-adaptive system, we know that there is a set of parameters that provides a good performance with low dynamics. In the explored scenario, this corresponds to Experiment e05, where the energy consumption shows an improvement with respect the figures obtained using a fixed transmission power set to the maximum and reaches a 99% PDR in just 11 iterations of the self-adaptive loop.

## 6. Discussion and Future Works

We have observed in the dynamic results of the system presented in the past section that even in the event of having a steady state regarding the communication range (expressed as no changes in the transmission power), the number of neighbors of each node is not the always the same. For instance, we can observe in Table 3 and in Figure 10c that Experiment e05 has the least number of changes in the transmission power of the deployed nodes. Additionally, if we look at Figure 11c, we will see that the number of neighbors registered in each node is not the same for all of them and also changes frequently. We can guess that this is due to the possible uncontrollable changes in the environment. Therefore, we can make the following proposition:

**Proposition 1.**  In an arbitrarily-deployed wireless sensor network, the node degree that guarantees the network connectivity is unbalanced.

We also have obtained results that show how there are configurations and conditions where the use of the proposed self-adaptive system improves both the connectivity and the energy consumption from the point of view of the whole network.

During the analysis of the system, we have observed other interesting areas where the proposed system can be improved and that we consider worth further exploration.

### 6.1. Future Works

The performance analysis for the proposed fuzzy control-based self-adaptive system has been done only in an outdoor scenario with just one arbitrary deployment. Other scenarios, either indoor or outdoor, should be explored in further works, including also different deployment schemes. The system’s performance results obtained should then be compared to the results shown in this paper.

Furthermore, in the proposed system, we have relied on the use of fuzzy functions for the decision-making blocks of the controller. These two functions operate in a similar way, as described in the system description: they both use one input and generate one output. It may be of interest to explore the use of more inputs, in particular in the case of the primary loop function of decision making. In this case, we were using the error on the node degree $e_{ND}$ as an input once it has been normalized as $e_{1}$. If for instance we add the previous communication range variation rate (Δcr(k-1)) as an input, could it be used to help in the reduction of the oscillations? If so, could this eliminate the need for the use of a tolerance value like $\xi_{ND}$?

As we are talking about the functions of decision-making, although we have explored the use of fuzzy logic-based functions, the self-adaptive system is open to the use of any other kind of function or mechanism for this task. For instance, it can be worth exploring the use of genetic and evolutionary algorithms for decision-making [35], multi-criteria decision-making methods [36] or any other one. The performance and system impact for these other functions should also be analyzed.

The analysis presented in this paper has also provided a configuration for the parameters of the self-adaptive system that give really good performance results. However, we cannot affirm that this configuration is the best one or even if there is a best one. The use of machine learning techniques to identify the best or the fittest parameter set is also another path for further research. It can also be done taking into account that the configuration can be different for each node in the network, depending on its own context and environmental conditions.

Finally, we have noticed that there are oscillations in the node degree even when there is no change in the transmission power of any node in the network. When this oscillation is within the tolerance parameter, the network can remain in the steady state. However, there is a chance that this oscillation can introduce errors in the system, causing it to enter into an active adaptation cycle. We have considered that these oscillations in the node degree can be due to changes in the environmental conditions. Additionally, these changes cause more oscillations with respect of those nodes that are located in the limit of the communication range of their neighbors. The following subsection proposes an idea of a possible workaround to cope with this problem.

#### 6.1.1. Detailed Description of the Proposal for Further Exploration of the Use of Weighted Links

The proposed fuzzy control-based self-adaptive system has been defined to use the node degree as a reference to make an estimated adjustment of the transmission power in order to achieve a minimum connectivity represented by the expression k-connected. The results analyzed in this paper and already discussed show that the node degree is a value that changes even when there is no change in the network parameters. This happens because some nodes can and usually will be located in a frontier area with respect to a reference node. Thus, a minor change in the environment conditions can cause their connectivity to the reference node to be interment.

**Figure 16:**
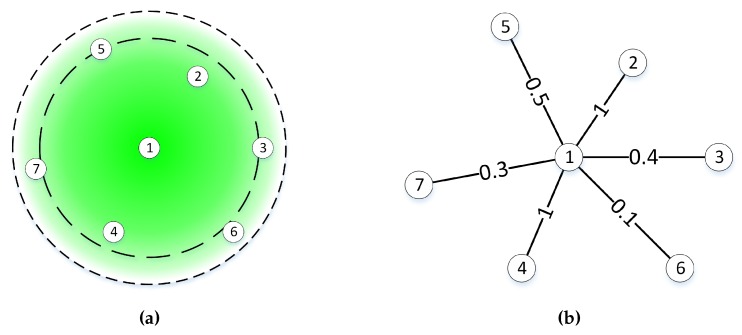
Use of link weight. (**a**) Communication range model; (**b**) communication range model weighted graph.

Just take a look at Figure 16a. In this figure, we have depicted a potential scenario. The reference node is labeled as “1”, and a disc communication range approach is used for explaining the theory. There are two discs in the figure. The inner disc represents the area where the connectivity is not affected by the environment. Of course, the quality of the communication can and will vary, but the nodes inside this disc will always appear as neighbors to Node “1”. The outer disc represents the limit of the communication range for Node “1”, and the ring between the inner and the outer discs is the area of the nodes that can be neighbors or not at different times.

If we just use the same probabilistic approach as defined for the system under test, Node “1” can have any number from two to six neighbors at any time. If the $ND_{R}$ and the $\xi_{ND}$ values are selected, two neighbors are not enough to guarantee the network connectivity; we can assure that there will be iterations of the control loop where the transmission power will be readjusted. Additionally, in a case like the one proposed, a readjustment of the transmission power may not be necessary, or even worse, can introduce new variations on the environment breaking the steady state of the networks.

Therefore, it could be interesting to explore the use of other probabilistic approaches, like considering the quality of the communication of every potential neighbor besides the node degree. For instance, in Figure 16, we have assigned a value between 0.0 and 1.0 to each potential neighbor. In this example, the value represents the probability that the node will be seen as a neighbor from Node “1”. We can then sum the weights, so we can say that the connectivity quality of the neighborhood for Node “1” is 3.4.

## Figures and Tables

**Table 1:** Rule analysis for the oscillations rule. NB, negative big; NS, negative small; ZV, zero value; PS, positive small; PB, positive big; NV, negative value; PV, positive value; NC, negative change; ZC, zero change; PC, positive change.

		$e_{\mathrm{ND}}\left(k-1\right)$								
		$e_{\mathrm{ND}}(k)=\mathrm{NV}$			$e_{\mathrm{ND}}(k)=\mathrm{ZV}$			$e_{\mathrm{ND}}(k)=\mathrm{PV}$		
		${\mathrm{ND}(k)>\mathrm{ND}}_{R}$			$\mathrm{ND}(k)\approx {\mathrm{ND}}_{R}$			${\mathrm{ND}(k)<\mathrm{ND}}_{R}$		
		NV	ZV	PV	NV	ZV	PV	NV	ZV	PV
	**NB**	NC	NC	NC	ZC	ZC	ZC	PC	PC	PC
	**NS**	NC	NC	NC	ZC	ZC	ZC	ZC	PC	PC
**Δ*cr(k−1)***	**ZV**	ZC	NC	NC	ZC	ZC	ZC	PC	PC	ZC
	**PS**	NC	NC	ZC	ZC	ZC	ZC	PC	PC	PC
	**PB**	NC	NC	NC	ZC	ZC	ZC	PC	PC	PC

**Table 2:** Parameters and results from previous work.

*Experiment*	${\mathrm{ND}}_{R}$	$\xi_{\mathrm{ND}}$	$k_{\mathrm{CR}}$	$P_{\mathrm{TX}}$ (dBm)	$J_{e}$ (mAh)	$J_{c}$
e01	—	—	—	−3	4283.19	1.2500
e02	—	—	—	−15	3408.00	13.7053
e03	2	0	1	—	3921.53	21.6875
e04	2	0	3	—	3947.76	12.0170
e05	2	1	3	—	3639.89	4.3095
e06	3	1	3	—	3817.46	7.0283
e07	3	0	3	—	3846.54	5.5803
e08	3	0	1	—	3865.22	10.3661
e09	3	1	1	—	3798.83	9.4375
e10	2	1	1	—	3952.63	24.5000

**Table 3:** Figure of merit for the communication range dynamics and PDR estimations.

Experiment	$J_{d}$	Rounds to 90%PDR	Rounds to 99% PDR	PDR at Round 30 (%)
e03	150	>30	>30	59.94
e04	211	>30	>30	76.40
e05	58	7	12	99.99
e06	83	25	>30	93.15
e07	205	16	>30	98.20
e08	182	24	>30	94.64
e09	127	22	>30	96.14
e10	84	>30	>30	42.94

**Table 4:** Evaluation of the effects of each input parameter for energy consumption.

Parameter Level	${\mathrm{ND}}_{R}$	$\xi_{\mathrm{ND}}$	$k_{\mathrm{CR}}$
**$SN_{p1}$**	3865.4525	3895.2625	3884.5525
**$SN_{p2}$**	3832.0125	3802.2025	3812.9125
**$\Delta J_{e}$**	**33.44**	**93.06**	**71.4**

**Table 5:** Evaluation of the effects of each input parameter for the connectivity.

Parameter Level	${\mathrm{ND}}_{R}$	$\xi_{\mathrm{ND}}$	$k_{\mathrm{CR}}$
**$SN_{p1}$**	25.8917	21.0376	29.0041
**$SN_{p2}$**	15.8518	20.7059	12.7394
**$\Delta J_{e}$**	**10.0398**	**0.3317**	**16.2647**

**Table 6:** Figure of merit for communication range dynamics.

Parameter Level	${\mathrm{ND}}_{R}$	$\xi_{\mathrm{ND}}$	$k_{\mathrm{CR}}$
**$SN_{p1}$**	125.75	187	135.75
**$SN_{p2}$**	149.25	88	139.25
**$\Delta J_{e}$**	**23.5**	**99**	**3.5**

## Abbreviations

The following abbreviations are used in this manuscript:

AODV: *Ad hoc* On-demand Distance Vector
FDM1: Function of Decision Making for the primary loop
FDM2: Function of Decision Making for the secondary loop
LQI: Link Quality Indicator
LQRP: Link Quality Routing Protocol
MAPE-K: Monitor, Analyze, Plan, Execute, Knowledge
PDR: Packet Delivery Ratio
RSSI: Received Strength Signal Indicator
SunSPOT: Sun Small Programmable Object Technology
TM1: Monitor Task for the primary loop
TM2: Monitor Task for the secondary loop
TR1: Reasoner Task for the primary loop
TR2: Reasoner Task for the secondary loop
TA1: Actuator Task for the primary loop
UML: Unified Modeling Language
WSAN: Wireless Sensor and Actuator Networks
